# Editorial: Fatigue in Multiple Sclerosis

**DOI:** 10.3389/fneur.2015.00266

**Published:** 2015-12-22

**Authors:** Christian Dettmers, John DeLuca

**Affiliations:** ^1^Kliniken Schmieder Konstanz, Konstanz, Germany; ^2^Neuropsychology and Neuroscience Laboratory, Kessler Foundation, West Orange, NJ, USA

**Keywords:** fatigue, fatigability, multiple sclerosis, cognition, functional imaging, pathophysiology, treatment, sleep disorders

**The Editorial on the Research Topic**

**Fatigue in Multiple Sclerosis**

Fatigue in Multiple Sclerosis (MS) is one of the most debilitating symptoms in patients with Multiple Sclerosis (pwMS). It interferes significantly with career as well as participation in everyday life activities. It is an enormous burden to the pwMS, his/her family and friends. Direct and indirect costs are extraordinary, both financial and psychosocial. Ongoing scientific studies struggle to understand the pathophysiology of fatigue in the hope of improving options for treatment. The past decade has seen much progress with important developments emerging to understand different phenomena related to fatigue. Most helpful and essential when discussing fatigue is the distinction between subjective sensations and objective changes in performance (1) and between trait and state fatigue (2). This Research Topic brings together ten novel and exciting perspectives written by leading authorities in this area from around the world from both clinical and scientific perspectives to understand the multidimensional nature of fatigue.

The first two chapters propose some new and unique concepts in trying to explain fatigue. *Dobryakova, Genova, DeLuca, and Wylie* summarize various lines of evidence suggesting that dopamine imbalance plays a major role in developing fatigue (Dobryakova et al.). The model builds upon an earlier framework for studying fatigue suggested by Chaudhuri and Behan (3, 4), who suspected that central fatigue was a “failure of the non-motor functions of the basal ganglia.” The current manuscript reviews the structural and functional neuroimaging evidence as well as pharmacological studies that suggest the critical role that dopamine plays in fatigue.

*Hanken, Eling, and Hildebrandt* focus on different aspects in their model (Hanken et al.). They suggest that the subjective feeling of fatigue is related to inflammation and increased levels of cytokines such as interleukin-1 (IL-1), IL-6, TNF-alpha. These inflammatory substances cause a sickness behavior – described as a highly organized strategy of an organism to cope with the infection. These authors refer to the original description of the sickness behavior by Maes et al. (5). From their structural imaging study, the authors concluded that structural alterations of the brain related to fatigue may be found in the insula, anterior cingulate cortex, and the hypothalamus. These structures are related to homeostasis and representation of internal bodily states.

Intuitively one might expect that the cognitive load has a significant impact on fatigue. Interestingly, *Sandry, Genova, Dobryakova, DeLuca, and Wylie* describe that it is not the cognitive load, but rather the length of the task (i.e., time on task) that is the major driving force in developing fatigue (Sandry et al.). This is intriguing and has practical implications for organizing daily routine and workloads in pwMS. They also raise the question, whether a decrease in information processing efficiency is the major obstacle or whether working memory is of equal importance, and also discuss whether fatigue is domain specific.

*Lukoschek, Sterr, Claros-Salinas, Gütler, and Dettmers* compare fatigue in a large population of pwMS and stroke patients using the vitality index of the SF-36. Normalized vitality scores in pwMS and stroke were clearly lower than in healthy controls (Lukoschek et al.). Fatigue was higher in pwMS than in stroke patients. Both patient groups showed no positive correlation between physical functioning and fatigue. Fatigue correlated with the working capacity in pwMS, but not in stroke patients. This work shows the dramatic impact of fatigue on pwMS.

*Sehle, Vieten, Mündermann, and Dettmers* elaborated on the objective assessment of motor fatigue (Sehle et al.). In a previous paper, they demonstrated that the attractor is a sensitive tool to describe variation and variability in gait patterns (6). This allowed for sensitive discrimination between pwMS with and without fatigue (7). Beside its relevance for assessment of motor fatigue this tool may serve as a model for the organic component of cognitive fatigue as “activity dependent loss of function” [analog to “activity dependent conduction block” (8)].

While motor fatigue represents a well-characterized concept of organic fatigue, fatigue is clearly multidimensional. *Schreiber, Lang, Kiltz, and Lang* elaborate on personality traits, disease coping, anxiety and depression, and their interaction (Schreiber et al.). It is not a question of either organic or psychogenic factors in describing fatigue, but in most instances, both factors contribute to the expression of fatigue. The authors suggest that fatigue in initial stages of MS might be largely driven by factors associated with disease coping, while in later stages inflammatory processes and lesions might dominate.

Although sleep disturbances are recognized as a cause of secondary fatigue, and although one might intuitively consider sleep disturbance as a contributing factor to fatigue, its prevalence, nature, and importance in patients suffering from fatigue are widely under-represented. *Strober* summarizes her own data and the literature regarding the contribution of sleep disturbance to the expression of fatigue (Strober).

The following chapters address pharmacological and non-pharmacological interventions in pwMS. Disease modifying drugs are generally used to reduce relapses and progression. *Kunkel, Fischer, Faiss, Daehne, Köhler, and Faiss* describe the effect of Natalizumab on cognition, fatigue. and depression in a longitudinal, observational study that spanned a 2-year period (Kunkel et al.). They found significant improvements in attention and depression after this period.

*Penner, Sivertsdotter, Celius, Fuchs, Schreiber, Berkö, and Svenningsson* raised a similar issue (Penner et al.). In a previous study, they described the improvement of total, motor, and cognitive fatigue during treatment with Natalizumab and 1-year follow-up. In the present chapter, they analyze the relationship between fatigue depression and daytime sleepiness. They found a close relationship between all three variables without being able to establish a causal relationship.

*Khan, Amatya, and Galea* completed the collection with a clinical summary on the management of fatigue in pwMS (Khan et al.). Treatment options include non-pharmacological interventions such as multi-disciplinary rehabilitation, specific rehabilitation interventions, and physical modalities such as exercise, aquatic therapy, Tai chi, cooling devices among others. Behavioral and educational interventions are also assessed, including fatigue management programs, energy conservation programs, mindfulness-based interventions, and cognitive and psychological interventions. Pharmacological interventions are reviewed as well and the evidence levels are summarized.

This Research Topic represents the first attempt to provide novel and the most up-to-date clinical, psychological, and physiological data related to fatigue. It is a “must” for every clinician, neurologist, and psychologist dealing with pwMS and/or fatigue.

## Conflict of Interest Statement

The authors declare that the research was conducted in the absence of any commercial or financial relationships that could be construed as a potential conflict of interest.
